# A randomized, multicenter, phase III study of gemcitabine combined with capecitabine versus gemcitabine alone as first-line chemotherapy for advanced pancreatic cancer in South Korea

**DOI:** 10.1097/MD.0000000000005702

**Published:** 2017-01-10

**Authors:** Hee Seung Lee, Moon Jae Chung, Jeong Youp Park, Seungmin Bang, Seung Woo Park, Ho Gak Kim, Myung Hwan Noh, Sang Hyub Lee, Yong-Tae Kim, Hyo Jung Kim, Chang Duck Kim, Dong Ki Lee, Kwang Bum Cho, Chang Min Cho, Jong Ho Moon, Dong Uk Kim, Dae Hwan Kang, Young Koog Cheon, Ho Soon Choi, Tae Hyeon Kim, Jae Kwang Kim, Jieun Moon, Hye Jung Shin, Si Young Song, Korean Society of Gastrointestinal Cancer

**Affiliations:** a Department of Internal Medicine, Institute of Gastroenterology, Yonsei University College of Medicine, Seoul; b Department of Internal Medicine, Catholic University of Daegu School of Medicine, Daegu; c Department of Internal Medicine, Dong-A University College of Medicine, Busan; d Department of Internal Medicine and Liver Research Institute, Seoul National University College of Medicine; e Department of Internal Medicine, Korea University College of Medicine; f Department of Internal Medicine, Gangnam Severance Hospital, Yonsei University College of Medicine, Seoul; g Department of Internal Medicine, Keimyung University School of Medicine; h Division of Gastroenterology and Hepatology, Department of Internal Medicine, Kyungpook National University School of Medicine, Daegu; i Digestive Disease Center and Research Institute, Department of Internal Medicine, Soon Chun Hyang University School of Medicine, Bucheon and Seoul; j Department of Internal Medicine, Pusan National University Hospital, Busan.; k Departments of Internal Medicine, Pusan National University Hospital, Yangsan; l Department of Internal Medicine, Digestive Disease Centre, Konkuk University School of Medicine, Seoul; m Departments of Internal Medicine, Hanyang University College of Medicine, Seoul; n Department of Internal Medicine, School of Medicine, Wonkwang University, Iksan; o Department of Internal Medicine, The Catholic University of Korea College of Medicine, St. Mary's Hospital; p Biostatistics Collaboration Unit, Medical Research Center, Yonsei University College of Medicine, Seoul, Korea.

**Keywords:** capecitabine, gemcitabine, overall survival, pancreatic cancer, progression-free survival

## Abstract

**Background:**

This phase III trial compared the efficacy and safety of gemcitabine plus capecitabine (GemCap) versus single-agent gemcitabine (Gem) in advanced pancreatic cancer as first-line chemotherapy.

**Methods:**

A total of 214 advanced pancreatic cancer patients were enrolled from 16 hospitals in South Korea between 2007 and 2011. Patients were randomly assigned to receive GemCap (oral capecitabine 1660 mg/m^2^ plus Gem 1000 mg/m^2^ by 30-minute intravenous infusion weekly for 3 weeks followed by a 1-week break every 4 weeks) or Gem (by 30-minute intravenous infusion weekly for 3 weeks every 4 weeks).

**Results:**

Median overall survival (OS) time, the primary end point, was 10.3 and 7.5 months in the GemCap and Gem arms, respectively (*P* = 0.06). Progression-free survival was 6.2 and 5.3 months in the GemCap and Gem arms, respectively (*P* = 0.08). GemCap significantly improved overall response rate compared with Gem alone (43.7% vs 17.6%; *P* = 0.001). Overall frequency of grade 3 or 4 toxicities was similar in each group. Neutropenia was the most frequent grade 3 or 4 toxicity in both groups.

**Conclusion:**

GemCap failed to improve OS at a statistically significant level compared to Gem treatment. This study showed a trend toward improved OS compared to Gem alone. GemCap and Gem both exhibited similar safety profiles.

## Abstract 2

Supplemental Digital Content is available in the text

## 1 Introduction

Pancreatic cancer is the fourth leading cause of cancer death in the United States^[1]^ and is still one of the most lethal malignancies with a 5-year survival of only 5%.^[2]^ In South Korea, approximately 5400 people develop exocrine pancreatic cancer each year, and the majority of patients present with inoperable advanced pancreatic cancer at the time of diagnosis.^[3,4]^

Gemcitabine (Gem) has been administered as a standard single-agent therapy for the treatment of advanced pancreatic cancer, with significant survival benefit compared with 5-fluorouracil (5-FU).^[5]^ Recently, the combination of fluorouracil, irinotecan, oxaliplatin, and leucovorin (FOLFIRINOX) and gemcitabine plus albumin bound paclitaxel particles have been associated with a significant improvement in overall survival (OS).^[6,7]^ However, owing to their greater toxicity, adoption of these treatment regimens is considered for patients with good performance status who do not have coexisting conditions.^[8,9]^

Capecitabine (Cap) is an orally administered, tumor-selective fluoropyrimidine carbamate that can be incorporated into schedules that provide prolonged fluorouracil exposure at lower peak concentrations, thus simulating continuous infusion of fluorouracil. Cap has a different mechanism of action than Gem with no overlapping toxicities. In addition, orally administered Cap chemotherapy is more convenient than intravenously administered 5-FU. The improved tolerability and similar efficacy of Cap compared with intravenous FU, and the convenience of oral administration make Cap an attractive treatment option in various other cancers.^[10,11]^

Recent advances in the management of advanced pancreatic cancer include 2 randomized phase III clinical trials indicating a response benefit associated with GemCap combination chemotherapy compared with Gem monotherapy.^[10,11]^ Cunningham et al^[11]^ reported that GemCap chemotherapy significantly improved the objective response rate (ORR) and was associated with a trend toward improved OS (hazard ratio [HR], 0.86; *P* = 0.08) as compared with treatment by Gem alone. Until now, no phase III study has been conducted to evaluate the efficacy of GemCap chemotherapy in an Asian patient population. To our knowledge, this study is the first phase III study conducted in an Asian patient population with advanced pancreatic cancer aimed at evaluating the efficacy and safety of GemCap combination therapy compared with Gem monotherapy.

## 2 Materials and methods

### 2.1 Patients

**Figure 1:**
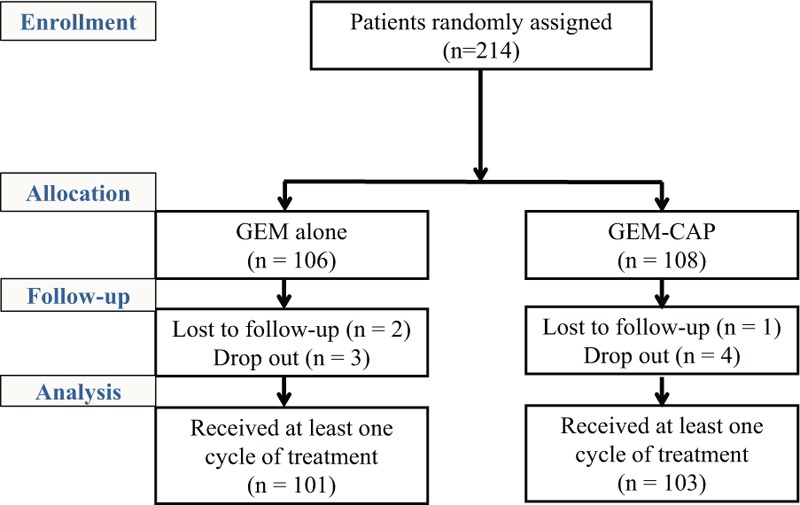
Study population assigned to treatment from 16 hospitals. For the statistical analysis, we finally selected 214 patients with pancreatic cancer who received GemCap chemotherapy or Gem chemotherapy alone. Gem = gemcitabine, GemCap = gemcitabine plus capecitabine.

A total of 214 advanced pancreatic cancer patients were enrolled from 16 hospitals in South Korea between 2007 and 2011. Patients were randomly assigned to GemCap or Gem treatment groups (Fig. 1). The eligibility of patients was confirmed histologically or with computed tomography (CT), magnetic resonance imaging (MRI), and positron emission tomography (PET) imaging. The inclusion criteria were inoperable locally advanced or metastatic pancreatic cancer according to the national comprehensive cancer network guidelines,^[9]^ no prior history of chemotherapy or radiotherapy, between the ages of 18 and 85 years, Eastern Cooperative Oncology Group (ECOG) performance status of 0 to 2, and adequate bone marrow, hepatic, and renal function. Exclusion criteria were pancreatic cancer other than adenocarcinoma, concurrent malignancy, brain metastasis, serious uncontrollable medical conditions, and significant cardiac history. The study protocol conformed to the ethical guidelines of the 1975 Helsinki Declaration and was approved by the Institutional Review Board of our institute. All recruited patients signed a written consent form before inclusion to the study. The present study is not registered in a clinical trials registry, since the present study was initiated before this became standard practice in South Korea.

### 2.2 Study protocol

This study was a multicenter, randomized, phase III trial with OS as the primary endpoint, conducted in Asian patients with advanced pancreatic cancer. Secondary endpoints were progression-free survival (PFS), ORR, disease control rate, and toxicity. We randomly assigned eligible patients to each treatment arm on a 1:1 basis according to a computer-generated variable-size blocked randomization method. Randomization was stratified by extent of disease (locally advanced stage vs metastatic stage). GemCap arm received oral capecitabine 1660 mg/m^2^ daily for 3 weeks followed by a 1-week break plus Gem 1000 mg/m^2^ by 30-minute intravenous infusion weekly for 3 weeks every 4 weeks. Gem arm received 30-minute intravenous infusion weekly for 3 weeks every 4 weeks. The body surface area (BSA) was recalculated and the dose levels of GemCap were adjusted based on the recalculated BSA prior to each cycle. This treatment was continued until response evaluation criteria in solid tumors (RECIST)-defined progressive disease and cumulative toxic effects were developed, or if the patients chose to discontinue the treatment.^[12]^ A research nurse or doctor in every center checked the toxicities of the registered medication in this study. Regarding the follow-up schedule, all patients were administered treatment on days 1, 8, and 15 every 4 weeks in subsequent cycles. During hospitalization, all patients’ vital sign, laboratory results, and general condition were investigated. After discharge from hospital, all patients were followed up within 1 to 2 weeks to check their general condition.

### 2.3 Response evaluation

The ORR was defined as the proportion of patients with complete responses (CR) and partial responses (PR) as measured by spiral CT. OS was calculated from the date of diagnosis to the date of death. The response to treatment was evaluated by CT or MRI after every 2 cycles of chemotherapy were completed. The objective tumor response was evaluated according to the RECIST criteria (version 1.1). The levels of carbohydrate antigen (CA) 19-9 and chorioembryonic antigen (CEA) were measured at the end of every 2 cycles. Toxicities were evaluated according to the National Cancer Institute Common Toxicity Criteria, version 4.0.^[13]^

### 2.4 Statistical analysis

The sample size was estimated based on the hypothesis that the GemCap combination therapy would increase the median OS time by 2 months compared with single-agent Gem (overall type I error possibility, alpha of 0.05 and power of 90%).^[10]^ The calculated sample size was 178 patients (89 patients per group). The 1 interim analysis was performed after observing approximately 124 (one-half) events. Statistical considerations were reviewed and discussed with Department of Biostatistics in Yonsei University College of Medicine. Data was expressed as mean ± standard deviation, n (%), or n, as appropriate. Variables were compared using the*χ*^*2*^ test for categorical data and Student *t* test for continuous variables to evaluate statistical significance of the differences in baseline characteristics between groups. Both OS and PFS estimates were calculated using the Kaplan–Meier method, and the survival curves were compared between treatment arms using the log-rank test. A *P* value of <0.05 was considered to indicate statistical significance. Statistical analyses were performed using SPSS version 18.0 (SPSS, Chicago, IL).

## 3 Results

### 3.1 Patient characteristics

The Median age was 54 years (range, 37–85 years) and the gender distribution was 120 men and 94 women. Patients were randomly allocated to Gem (n = 106) and GemCap (n = 108) groups. Ten (4.6%) patients were ineligible because of loss to follow-up or dropout from the study. These patients were included in the analyses on an intention-to-treat basis. Patient characteristics are shown in Table 1. Variables were well balanced between 2 groups.

**Table 1:**
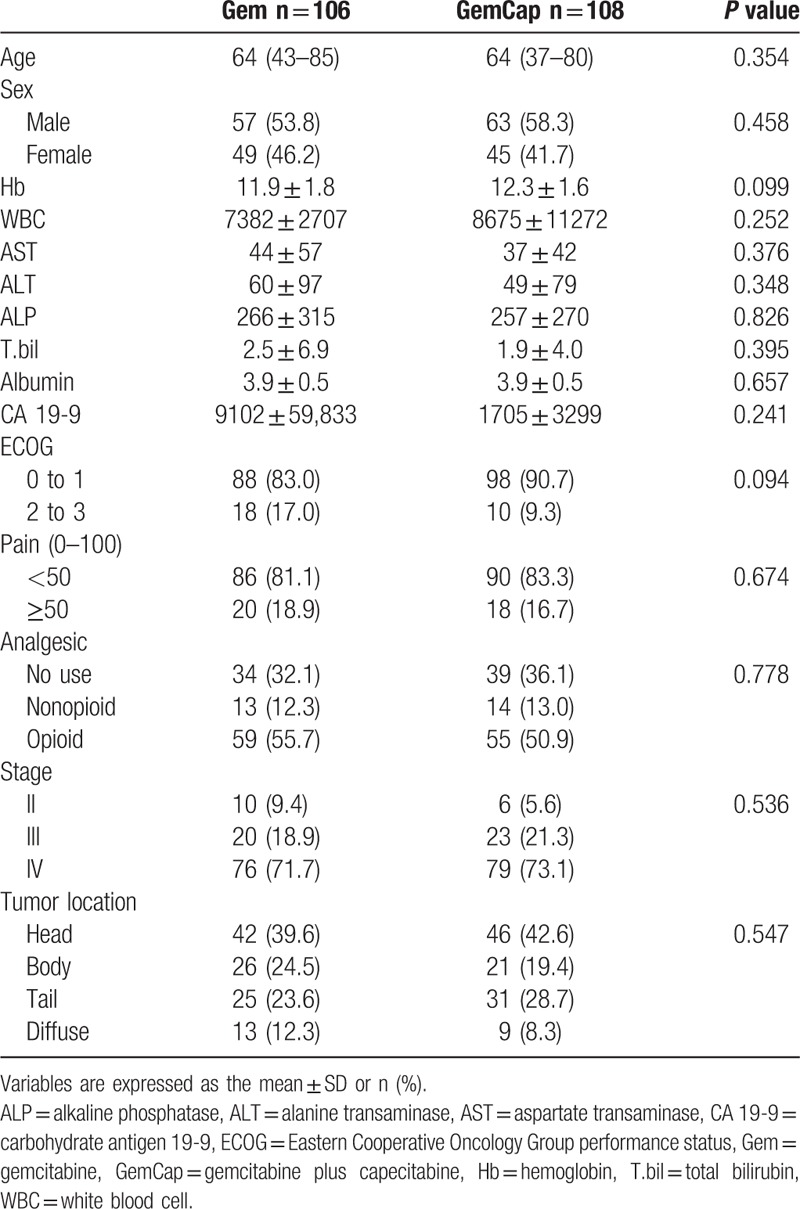
Baseline characteristics of patients.

### 3.2 Overall survival and progression-free survival

**Figure 2:**
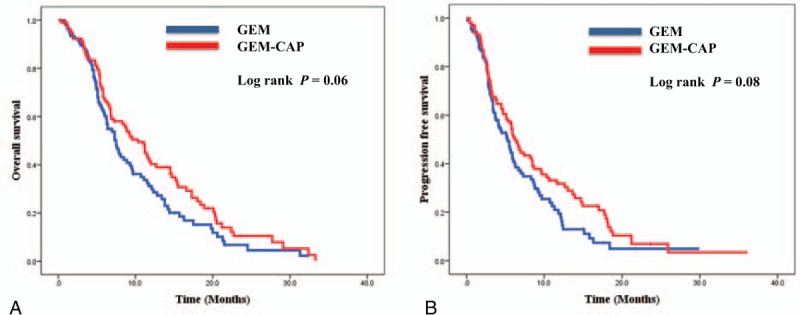
Kaplan–Meier overall survival curves and progression-free survival curves in pancreatic cancer patients. A, Median overall survival (OS) time, the primary end point, was 10.3 and 7.5 months in the GemCap and Gem arm, respectively (*P* = 0.06). B, Progression-free survival (PFS) was 6.2 and 5.3 months in the GemCap and Gem arm, respectively (*P* = 0.08). Gem = gemcitabine, GemCap = gemcitabine plus capecitabine.

**Figure 3:**
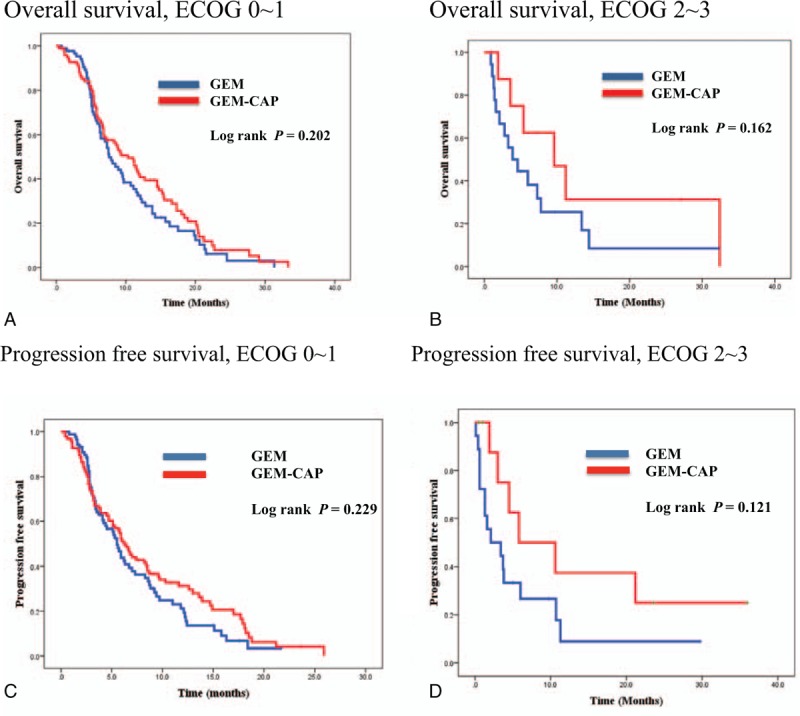
Kaplan–Meier overall survival curves and progression-free survival curves in patients according to performance status. Subanalysis in patients with good performance status did not show a significant prolongation of median OS time and PFS time in the GemCap arm compared with Gem arm. Gem = gemcitabine, GemCap = gemcitabine plus capecitabine, OS = overall survival, PFS = progression free survival.

GemCap was associated with a trend toward better OS compared with Gem alone (HR, 0.82; 95% CI, 0.67–1.01; *P* = 0.06). The median OS times for patients in GemCap and Gem treatment groups were 10.3 and 7.5 months, respectively. GemCap did not show a significantly improved PFS compared with Gem (HR, 0.87; 95% CI, 0.73–1.03; *P* = 0.08). The median PFS for GemCap was 6.2 months, and that for Gem was 5.3 months. The OS and PFS by treatment arm are indicated in Fig. 2. On subgroup analysis according to ECOG and age, there was no significant survival difference between the 2 groups (Fig. 3 and Supplementary figure).

### 3.3 Response to treatment

Patients randomly assigned to GemCap treatment group had a significantly improved ORR over those assigned to Gem (43.7% vs 17.6%, respectively; *P* = 0.001). The efficacies of the treatments are summarized in Table 2. Of the total 214 patients, none exhibited CR, 54 (25.2%) exhibited PR, and 58 (27.1%) were observed to have stable disease. Progressive disease occurred in the 66 patients (30.8%).

**Table 2:**
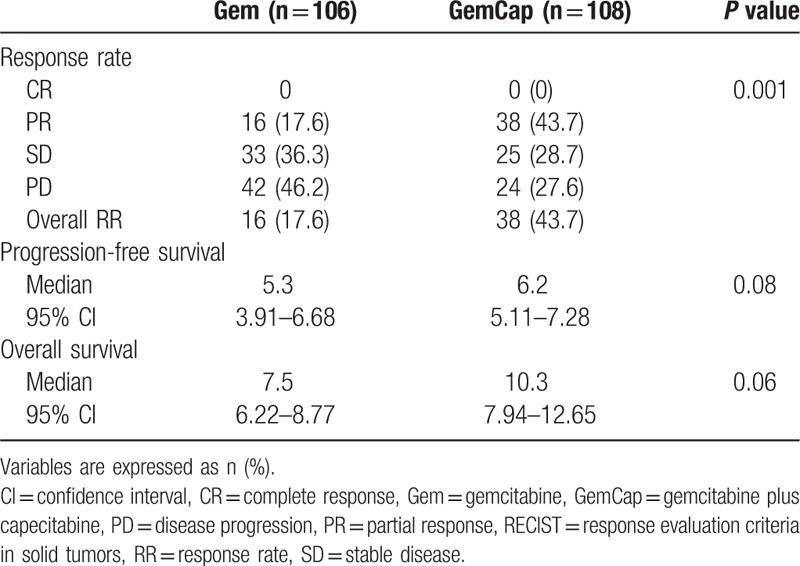
Response rates according to RECIST criteria.

### 3.4 Toxicities

Therapy-related toxicities were monitored in all 204 patients who were treated with 1000 mg/m^2^ Gem or 1660 mg/m^2^ of oral Cap. Both treatment arms were well tolerated. Table 3 summarizes the grade 3 or 4 toxicities associated with the treatment arms. The overall frequency of grade 3 or 4 toxicities was similar in each group. Neutropenia was the most frequently observed grade 3 or 4 toxicity in both groups (29.2%). Although GemCap treatment resulted in increased neutropenia compared with Gem treatment, febrile neutropenia was not observed more than Gem treatment. The frequencies of other adverse events such as nausea, vomiting, diarrhea, and stomatitis were similar between the 2 arms.

**Table 3:**
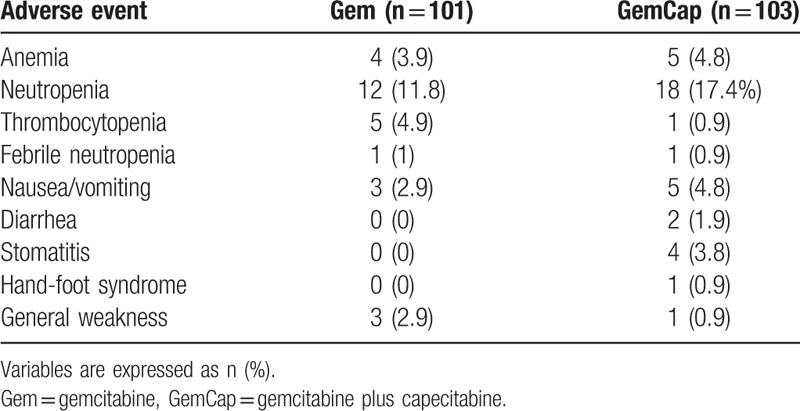
Grade 3 or 4 adverse events.

## 4 Discussion

This study did not show the statistical OS improvement in GemCap compared with Gem treatment in advanced pancreatic cancer patients. However, a trend toward improved OS in patients treated with GemCap was observed. This study also showed that advanced pancreatic cancer patients responded better to GemCap with acceptable levels of adverse events.

Pancreatic cancer remains as one of the most common and lethal cancers. FU-based chemotherapy was known to improve overall survival by approximately 3 months compared with best supportive care alone.^[8]^ In 1997, a study comparing Gem with FU in patients with advanced pancreatic cancer showed an improved overall survival of 1 month among patients receiving Gem.^[5]^ Over the next 10 years, multiple randomized studies comparing single-agent Gem with combination chemotherapy have been conducted showing no effective improvement in survival.^[14–17]^ Exceptionally, the incorporation of erlotinib, an EGFR inhibitor, resulted in a significant improvement of the overall survival by approximately 2 weeks (HR 0.82, *P* = 0.038, median 6.2 vs 5.9 months, respectively). However, owing to its limited effect and added toxicity, this treatment regimen has not been frequently adopted.^[18]^

Two recently conducted clinical trials showed prolonged overall survival of approximately 1 year in advanced pancreatic cancer patients. In the first study, more recent treatment regimens including FOLFIRINOX were associated with a significant improvement in median OS compared with Gem monotherapy (11.1 months vs 6.8 months, respectively; *P* <0.001).^[7]^ In the second study, Gem plus nanoparticle albumin bound-paclitaxel was also associated with a prolonged OS compared with Gem monotherapy (8.5 months vs 6.7 months, respectively; *P* <0.001).^[6]^ At present, these regimens are considered standard treatment for patients with good PS. However, treatment-related adverse events were also worsened with these regimens compared with Gem monotherapy, including grade 3/4 neutropenia (45.7% and 38%, respectively), and diarrhea (12.7% and 6%, respectively).

Therefore, alternative combination chemotherapeutic regimens have been studied for the treatment of patients with advanced pancreatic cancer. Among them, GemCap was identified as an alternative combination therapy for patients with advanced pancreatic cancer.^[19,20]^ In a randomized phase III trial, Cunningham et al^[11]^ showed a significant improvement in the PFS (HR, 0.78; 95% CI, 0.66–0.93; *P* = 0.004) and a trend towards improved OS (HR, 0.86; 95% CI, 0.72–1.02; *P* = 0.08) in patients receiving GemCap therapy compared with those receiving Gem monotherapy. In the study, Cunningham et al used intensive dose-combination regimen compared with another phase III trial (oral capecitabine 1660 vs 650 mg/m^2^/day; gemcitabine infusion on days 1, 8, and 15, every 4 weeks vs 1 and 8, every 3 weeks) and intensive dose-combination showed more effective results than another regimen.^[10,11]^ Using high dose of chemotherapy regimens, the present study of GemCap combination therapy produced significantly improved ORR of 43.7% and a median PFS of 6.2 months, respectively. These results were similar to previous phase II studies conducted in South Korea.^[20]^

To our knowledge, this study investigating the use of GemCap combination therapy for the treatment of advanced pancreatic cancer was a first in Asia. In this study, a longer OS of 10.3 months compared with previous phase III studies is reported. This is associated with the recent improvements in cancer supportive care such as pain control, complication control, and nutritional support. This data was also in agreement with previous phase II study data (10.3 months vs 10 months, respectively).^[20]^

There are some limitations in the study. We failed to show the significant difference in overall survival between GemCap and Gem due to an insufficient number of enrolled patients differing from the initially planned number. South Korea has universal health insurance system that covers almost the entire population. The medical insurance system of government did not cover the use of gemcitabine plus capecitabine on pancreatic cancer during the present study period. We could not prescribe additive gemcitabine plus capecitabine chemotherapy in patients with pancreatic cancer. And as a result, we did not enroll the sufficient number of patients during the study period. However, we showed a trend towards improved OS in patients who received GemCap although this was not statistically significant. Furthermore, response rates were significantly higher in GemCap patients compared with Gem (43.7% vs 17.6%, respectively). Second, the results from the new trials such as Von Hoff et al^[6]^ about the increased survival in pancreatic cancer were published in 2013. Unfortunately, it decreases the value and novelty of the results from the current study. However, there was no phase III study that has been conducted to evaluate the efficacy of GemCap chemotherapy in an Asian patient population until now.

Recently, many novel biological agents targeting pancreatic cancer and its microenvironment have been reported to be promising in preclinical investigations and phase I/II clinical studies.^[21–24]^ However, an effective therapeutic agent for the treatment of pancreatic cancer does not exist presently. Depending on patients’ health conditions, conventional chemotherapeutic agents are still administered.

In conclusion, this study was the first phase III study conducted in Asian patients using GemCap combination therapy. GemCap combination did not show statistically significant survival benefit compared with Gem treatment. There was a trend toward improved OS and better response in pancreatic cancer patients treated with GemCap.

## Acknowledgments

The authors are grateful to Department of Research Affairs, Biostatistics Collaboration Unit, Yonsei University College of Medicine, Seoul, Korea for the help with the Statistical Analysis.

## Supplementary Material

Supplemental Digital Content
