# Supplementary material for: A randomized, multicenter, phase III study of gemcitabine combined with capecitabine versus gemcitabine alone as first-line chemotherapy for advanced pancreatic cancer in South Korea
Source: Medicine (Baltimore). 2017 Jan 10;96(1):e5702. doi: 10.1097/MD.0000000000005702 (PMC5228666; doi:10.1097/MD.0000000000005702)

Supplementary Figure 1 Kaplan-Meier overall survival curves and progression free survival curves in patients according to patient age. Sub-analysis in patients did not show a significant prolongation of median OS time and PFS time in the GemCap arm compared to Gem arm.

Supplementary Figure 1

(A) Overall survival, Age ≤ 60

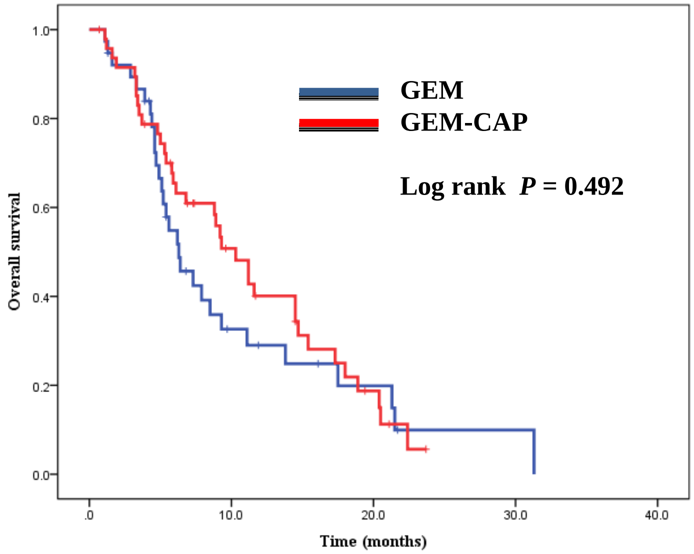

(B) Overall survival, Age > 60

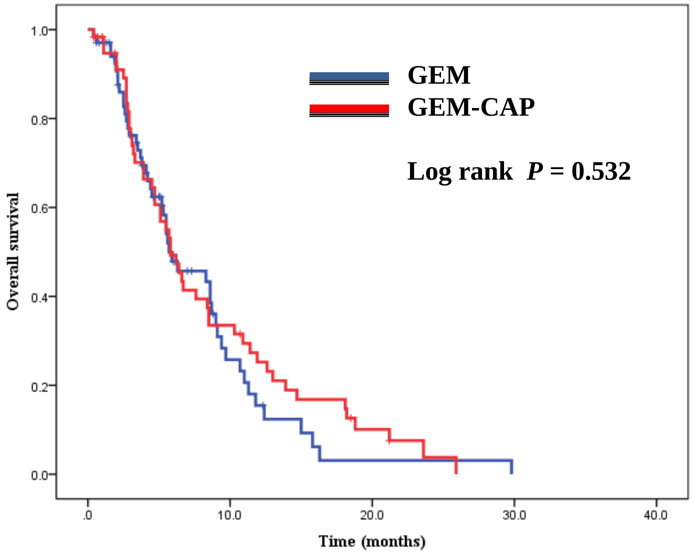

Supplementary Figure 1

(C) Progression free survival, Age ≤ 60

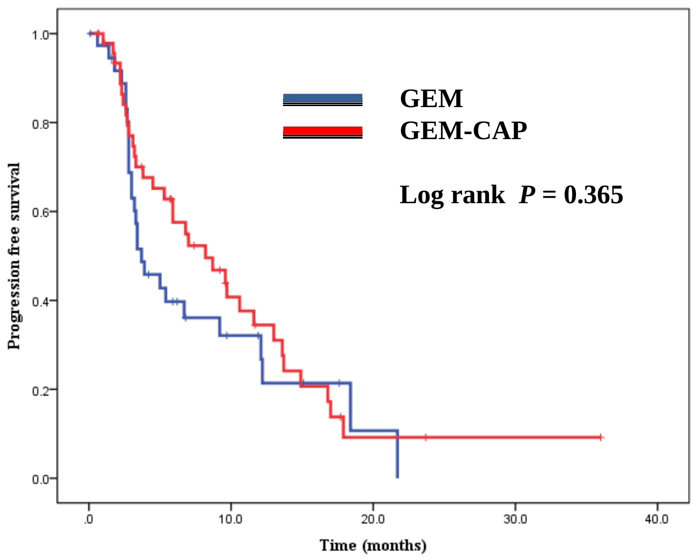

(D) Progression free survival, Age > 60

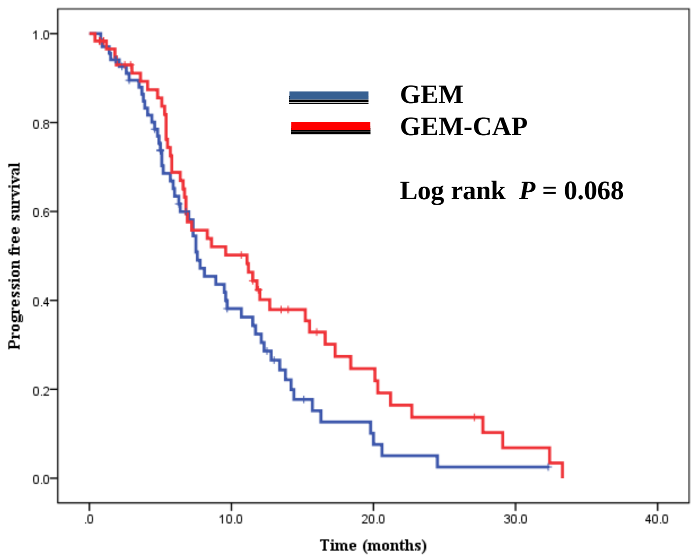

Supplement: Supplemental Digital Content [file medi-96-e5702-s001.pdf]
